# Effects of Methyl Farnesoate on the Growth and Antioxidant Capacity of *Neocaridina denticulata*

**DOI:** 10.3390/antiox14060635

**Published:** 2025-05-25

**Authors:** Ying Chen, Xiaojuan Sun, Jiahao Du, Jingjie Hu, Zhenmin Bao, Zhe Qu

**Affiliations:** 1 Key Laboratory of Tropical Aquatic Germplasm of Hainan Province, Sanya Oceanographic Institution, Ocean University of China, Sanya 572000, China; chenying5050@stu.ouc.edu.cn (Y.C.); sun19862703931@163.com (X.S.);; 2 MOE Key Laboratory of Marine Genetics and Breeding, and Shandong Key Laboratory of Marine Seed Industry (preparatory), Ocean University of China, Qingdao 266003, China

**Keywords:** sesquiterpenoids, ecdysteroid, endocrine disruptor, transcriptome, atyidae, crustacean, arthropods

## Abstract

Sesquiterpenoid hormones are widely present in arthropods and play crucial roles in growth, molting and reproduction. Methyl farnesoate (MF) functions similarly to juvenile hormone (JH) in crustaceans, playing a broad regulatory role in their growth and development. However, compared to insects, systematic studies on the mechanisms of sesquiterpenoid hormones in crustaceans are still lacking. *Neocaridina denticulata*, a small freshwater shrimp known for its fast growth, high reproductive capacity and ease of maintenance, is an ideal model organism for crustacean research. To investigate the effects of MF on the growth and development of juvenile *N. denticulata*, MF feeding experiments were conducted and the changes at the phenotypic and molecular levels were examined. In this experiment, the basal diet was used as a control, with 40 μg/kg, 4 μg/kg and 0.4 μg/kg of MF added to the feed. The MF-enriched diets were fed to juvenile *N. denticulata* and the growth in body length was measured every 10 days. After 40 days of feeding experiment, the activities of amylase (AMS), lipase (LPS), trypsin (Try), superoxide dismutase (SOD), malondialdehyde (MDA) and glutathione peroxidase (GSH-PX) were assessed, and transcriptome analysis was performed. We found that MF showed an initial inhibitory effect on body length (day 30), but by day 40, the low-concentration group exhibited significantly enhanced growth compared to the control, indicating a dose- and time-dependent effect. Activities of AMS, LPS, Try and SOD generally decreased, whereas MDA levels and GSH-PX activity increased after 40 days of MF exposure. Moreover, transcriptomic analysis revealed that MF regulated various biological processes including growth, metabolism and immune responses. High concentration group appeared to restrict growth via modulation of exoskeleton-related and cellular stress genes. Medium concentration group enhanced growth by optimizing metabolic and signaling pathways. Low concentration group preferentially up-regulated genes related to muscle function, potentially supporting locomotion and competitive ability. This study provides new insights into the regulatory mechanism of sesquiterpenoid hormones in crustaceans and their potential applications in aquaculture in the future.

## 1. Introduction

Hormones are key regulators of animal development, shaping both physiology and morphological diversity during evolution, and serving as critical endocrine messengers that coordinate complex regulatory networks to maintain systemic metabolic equilibrium [1]. Sesquiterpenoids and ecdysteroids constitute two representative hormone groups in arthropods [2]. Methyl farnesoate (MF), a juvenile hormone-like sesquiterpenoid hormone, was first identified in a crab in 1987 [3]. Currently, MF has been shown to be widely present in crustaceans and to participate in regulating key physiological processes such as reproduction, stress response, molting, growth and development [4].

In crustaceans, MF has a stimulatory effect on both female and male individuals. It not only stimulates vitellogenesis but also regulates male behavior [5]. Additionally, MF plays an important role in environmental adaptation and the regulation of physiological metabolism [6] including molting cycle [7]. Its specific effects, however, may vary based on species, sex, developmental stage and dosage [8]. Oral administration experiments indicated the key role of MF in growth and development [9,10], especially in larval stages [11]. Previous studies have shown that crustaceans exhibit a significant concentration-dependent response to exogenous hormones, with high concentrations potentially inducing oxidative stress or metabolic disturbances [12]. MF had a dose-dependent regulatory role in the molting and growth of crustaceans, but the underlying molecular mechanisms remain unclear [4,13].

*Neocaridina denticulata* is a freshwater shrimp species with significant economic and ecological value, and it also serves as an important model organism for studying the growth and development mechanisms of crustaceans [14,15]. To further elucidate the effects of MF dosage on the growth and development of crustacean larvae and its underlying molecular mechanisms, this study explored the impacts of different concentrations of dietary MF on the growth and development of juvenile *N. denticulata* from both molecular and phenotypic perspectives. Growth rate, digestive enzyme activities, antioxidant capacities and the regulatory effects on related gene expressions were comprehensively assessed. Findings of this study expanded our understanding of sesquiterpenoid hormone regulation on the growth and antioxidant capacity of decapod and provided a theoretical basis for optimizing shrimp aquaculture and environmental management.

## 2. Materials and Methods

### 2.1. Experimental Feed Preparation

The experimental feeds were prepared based on the method described previously [16], with the specific composition and nutrient content of the feeds (Table 1). Each feed component was pulverized with a grinder and sieved through a 300-mesh size nylon sieve, accurately weighed and thoroughly mixed. The preparation process was as follows: MF (Echelon Biosciences, Salt Lake City, UT, USA) was dissolved in ethanol to prepare a master batch of 1 mg/mL, which was then diluted to concentrations of 10^−9^, 10^−8^ and 10^−7^ mg/μL with distilled water. These MF solutions were then added to the feed (0.4 L/kg) to prepare a uniform, smooth dough containing 0.4 μg, 4 μg and 40 μg MF per kg of feed, respectively. The pellet feed was extruded into 1.0 mm diameter pellets using a double-sided knife pasta press. The prepared pellet feeds were dried in an oven at 60 °C to about 10% humidity, and the feeds were crushed into smaller particles. The control feed was not supplemented with MF, and all other preparations were identical to those of the treatment groups. All feeds were stored at −20 °C until experimental use.

### 2.2. Animals and Experiment Design

In this study, laboratory-bred *N. denticulata* were acclimatized to the culture system for one week prior to the experiment. The water temperature was maintained at 26 ± 0.5 °C, with a 14 h light/10 h dark photoperiod. During this period, water quality was controlled using an inbuilt recirculating filtration system, and the shrimps were fed a basal diet twice daily: in the morning (8:00 a.m.) and evening (8:00 p.m.). A total of 120 one-week-old juvenile shrimp were randomly divided into four groups. The control group (Juv_c) was fed the basal diet, while the experimental groups received diets supplemented with MF at concentrations of 10^−9^ (Juv_9), 10^−8^ (Juv_8) and 10^−7^ (Juv_7). The experiment lasted for 40 days, with the feed being quantitatively administered twice daily. Water quality was monitored regularly; body length data were recorded, and the length growth rate (LGR) was calculated (LGR (%) = (L_t_−L_0_)/L_0_ × 100%; L_t_ (µm): final body length; L_0_ (µm): initial body length). At the end of the experiment, whole animal samples from each group were collected and stored at −80 °C after flash-freezing in liquid nitrogen.

### 2.3. Digestive Enzyme and Oxidative Stress Indexes Determination

Indicators of digestive enzyme activity of amylase (AMS), lipase (LPS) and trypsin (Try) were examined. Indicators of antioxidant enzyme activity included superoxide dismutase (SOD) activity, malondialdehyde (MDA) content and glutathione peroxidase (GSH-PX) activity. After the tissue homogenate was obtained by grinding *N. denticulata*, the supernatant was centrifuged and separated for the determination of the above enzymes and indexes. Enzyme activity analyses were conducted using commercial assay kits (Jiancheng Biological Engineering Institute, Nanjing, China) according to the manufacturer’s instructions.

### 2.4. RNA Isolation, Library Construction and RNA-Seq Analysis

Total RNA was isolated from three randomly selected whole individuals of *N. denticulata* from each group (*n* = 3, 12 in total). These RNA samples were subjected to Illumina transcriptome library construction and sequencing at LC-Bio Technologies (Hangzhou, China). The raw data from Illumina sequencing were trimmed and quality filtered using Cutadapt v4.1 software [17]. De novo assembly of clean data was performed using Trinity v2.15 software to generate high-quality transcripts [18]. The integrity of transcriptome splicing was assessed using BUSCO [19]. Functional annotation of Unigenes was performed using DIAMOND v2.0.15 [20], aligning against six reference databases: NCBI_NR, GO, KEGG, Pfam, SwissProt and eggNOG. The expression levels of Unigenes were estimated using Salmon v1.9.0 [21] and normalized to TPM (transcripts per million) values to obtain mRNA expression profiles.

Differentially expressed genes (DEGs) were identified using DESeq2 [22] with a statistical significance threshold of |log2FC (fold change)| > 1 and *p* < 0.05. All DEGs were mapped to the Gene Ontology (GO) and Kyoto Encyclopedia of Genes and Genomes (KEGG) databases for comparison. Enrichment analysis was performed using the OmicStudio (https://www.omicstudio.cn/tool (6 February 2023), with a significance threshold set at *p* < 0.05, to elucidate the potential biological implications of DEGs.

### 2.5. Weighted Gene Co-Expression Network Analysis

A weighted gene co-expression network was constructed using the WGCNA package in R [23]. A soft-thresholding power (β = 6) was selected to approximate scale-free topology (R^2^ > 0.85). Pairwise gene correlations were transformed into an adjacency matrix, which was further converted into a topological overlap matrix (TOM) to minimize spurious connections. Hierarchical clustering with dynamic tree cutting (minimum module size = 200, detectCutHeight = 0.99, MEDissThres = 0.3) was applied to identify co-expression modules. Module eigengenes (MEs) were calculated as the first principal component of each module. To associate modules with MF hormone treatment, a hypergeometric test was implemented to evaluate the overlap between module genes and the DEG set. Modules with significant enrichment (*p <* 0.05, false discovery rate [FDR]-adjusted) were designated as hormone-responsive.

### 2.6. Quantitative Real-Time PCR Validation

To further validate the reliability of the RNA-seq data, the expression level of five DEGs, including *MFE*, *JHAMT*, *Met*, *PRDX3* and *CYP307A1*, was detected using quantitative real-time PCR validation (qPCR), with *EF-1α* as the internal reference gene. The primer sequences are provided in Table 2. qPCR was performed using ChamQ Universal SYBR qPCR Master Mix (Vazyme, Nanjing, China), and the reaction conditions were as follows: 95 °C, 30 s; 95 °C, 10 s; 60 °C, 30 s, 40 cycles. The generation of a melt curve by gradually increasing the temperature from 60 °C to 95 °C in 1 °C steps to confirm amplification specificity. The relative expression levels of DEGs were calculated using the 2^−∆∆Ct^ method [24].

### 2.7. Statistical Analysis

Significant differences among different groups were tested by one-way analysis of variance (ANOVA) using SPSS26.0 (IBM, Armonk, NY, USA). All data were expressed as mean ± standard deviation (mean ± SD). Duncan’s multiple-range test was used for multiple comparisons of group means. Differences were considered statistically significant at *p* < 0.05.

## 3. Results

### 3.1. Effect of Dietary MF on the Growth Performance of Juvenile N. denticulata

Different concentrations of MF had different effects on the length growth of *N. denticulata*, with the low concentration (Juv_9 group) significantly promoting growth, while the high concentration (Juv_7 group) continued to inhibit growth throughout the experiment (Table 3). The highest LGR of 13.22% was recorded in the Juv_9 group on day 20, while it was lower in the Juv_8 (9.37%) and Juv_7 (9.29%) groups. By day 30, the LGR of the Juv_7, Juv_8 and Juv_9 groups were 18.69%, 18.88% and 18.94%, respectively, all of which were lower than the control group (19.40%). However, on day 40, the highest LGR was observed in the Juv_9 group, and the lowest in the Juv_7 group (Table 3).

### 3.2. Changes in Digestive Enzyme Activity and Antioxidant Capacity

**Figure 1:**
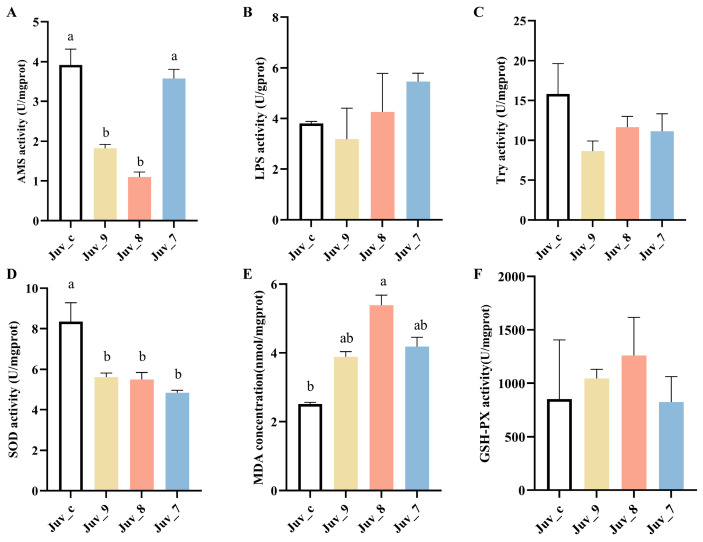
Digestive and antioxidant enzyme activities of juvenile *N. denticulata* after feeding MF for 40 days. (**A**) AMS; (**B**) LPS; (**C**) trypsin; (**D**) SOD activity; (**E**) MDA concentration; (**F**) GSH-PX activity. Different letters indicate significant differences (*p*< 0.05). All data are presented as the mean ± SD (*n* = 3).

Feeding different concentrations of MF produced significant effects on digestive and antioxidant enzyme activities of *N. denticulata* (Figure 1). AMS activity was highest in the control group, while Juv_9 and Juv_8 groups exhibited significantly lower activity than the control group. However, the high concentration in the Juv_7 group did not show a significant difference, indicating that low concentrations of MF had an inhibitory effect on AMS activity. The LPS and Try activities were not significantly changed in different groups, suggesting that MF had a minimal effect on these activities. In contrast, antioxidant enzyme activities were more significantly affected, with SOD activity being significantly reduced in MF dietary groups, suggesting that high MF intake may lead to a decrease in antioxidant capacity. Malondialdehyde (MDA) concentration was significantly higher in the Juv_8 group, indicating an increased level of lipid peroxidation and oxidative damage in *N. denticulata* after treatment with MF. Conversely, GSH-PX activity did not show significant differences among all treatment groups, suggesting that MF had no significant effect on GSH-PX activity. Overall, high concentrations of MF not only inhibited SOD activity but also significantly increased the MDA concentration, suggesting that sustained oxidative stress may have been triggered. Meanwhile, low concentrations of MF exerted a significant inhibitory effect on AMS activity, indicating that it may affect digestive function.

### 3.3. Transcriptomes and DEGs Analysis

A total of 562,324,770 raw reads were generated with an average of 46.8 M per sample. A total of 548,771,022 valid reads with high quality were obtained with an average of 45,730,918 for each sample after filtering (*n* = 3, 12 in total, Table S1).

**Figure 2:**
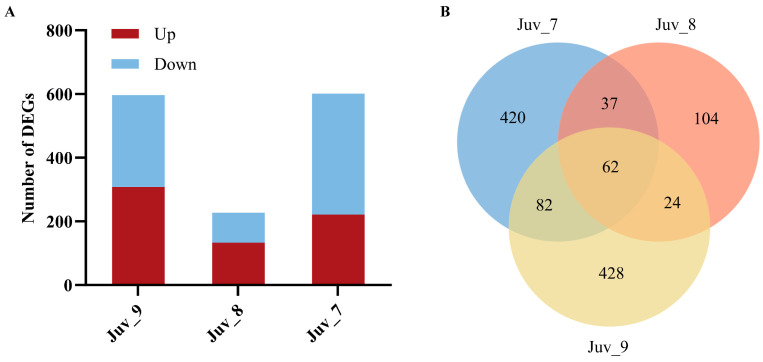
Effect of dietary MF on the gene expression of juvenile *N. denticulata*. (**A**) Numbers of DEGs in juvenile *N. denticulata* after different MF treatments; (**B**) Venn diagram of DEGs across samples.

Different concentrations of MF treatments significantly affected the gene expression of *N. denticulata* (Figure 2A). Compared with the control group (Juv_c), 601 DEGs (221 up-regulated and 380 down-regulated) were detected in the Juv_7 group; 227 DEGs (133 up-regulated and 94 down-regulated) were detected in the Juv_8 group; and 596 DEGs (308 up-regulated and 288 down-regulated) were detected in the Juv_9 group. The Venn diagram demonstrates the distribution and intersection of DEGs between different MF treatment groups (Juv_7, Juv_8, Juv_9) and the control group (Figure 2B). The Juv_7 group exhibited 420 specifically expressed DEGs, the Juv_8 group contained 104 specifically expressed DEGs, and the Juv_9 group showed 428 specifically expressed DEGs. These specific genes reflected the unique effects of different concentrations of MF treatment on gene expression. In addition, common DEGs were found among the treatment groups. A total of 62 DEGs were shared among the three treatment groups, which may be related to the core physiological regulatory functions of MF.

### 3.4. Functional Enrichment Analysis of DEGs

**Figure 3:**
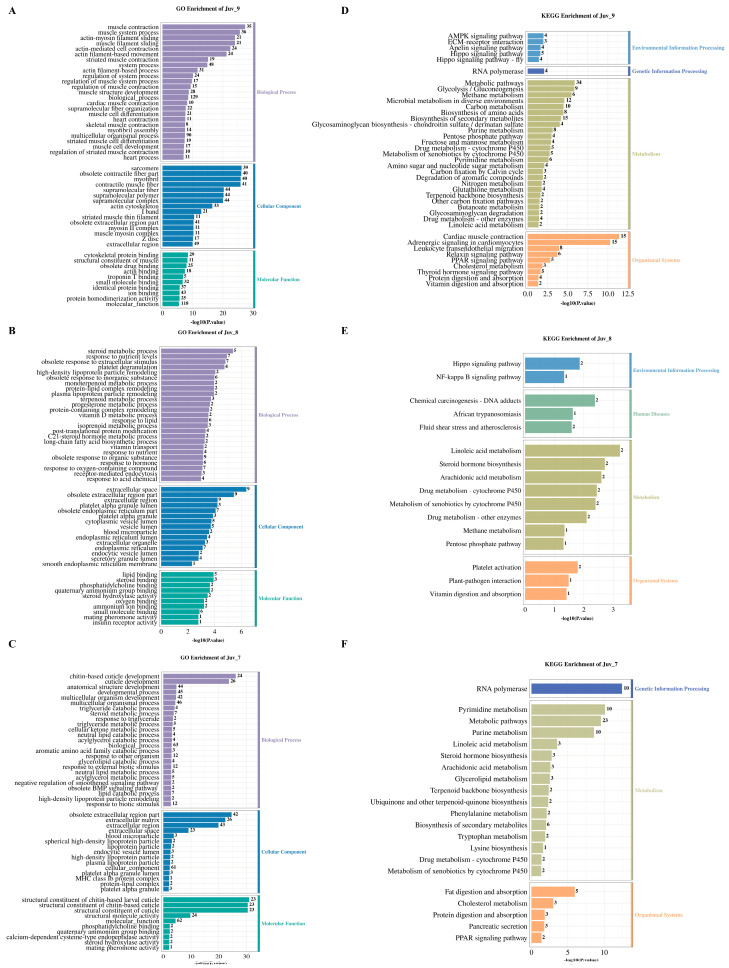
GO and KEGG enrichment analysis of DEGs in various MF feeding groups. (**A**–**C**) GO enrichment analysis chart for the MF treatment group at concentration of 10^−9^, 10^−8^ and 10^−7^; (**D**–**F**) KEGG enrichment analysis chart for the MF treatment group at concentration of 10^−9^, 10^−8^ and 10^−7^. The numbers on the bars indicate the number of DEGs.

GO enrichment analysis revealed that different concentrations of MF treatment significantly affected the functional classification of DEGs in *N. denticulata*. The Juv_9, Juv_8 and Juv_7 groups were significantly enriched for 1058, 503 and 361 GO terms, respectively (*p <* 0.05, Supplementary Table S2). Figure 3A–C illustrates the results of GO enrichment analysis, presenting the top 25 most enriched terms in Biological Process, the top 15 terms in Cellular Component and the top 10 terms in Molecular Function. The top enriched terms for each group can be read based on the maximum number on the bar.

KEGG pathway enrichment analysis revealed that the DEGs in the Juv_9, Juv_8 and Juv_7 groups were significantly enriched in 71, 16 and 27 pathways, respectively (*p* < 0.05; Figure 3D–F, Supplementary Table S3), demonstrating differences in functional properties caused by different MF dosages. All of these significantly enriched pathways were primarily associated with metabolism and organismal systems. All groups showed significant enrichment in “metabolism of xenobiotics by Cytochrome P450”, “Drug metabolism-cytochrome P450” and “Linoleic acid metabolism pathways”. Steroid hormone biosynthesis and arachidonic acid metabolism were shared between Juv_7 and Juv_8, whereas Juv_7 and Juv_9 exhibited common enrichment in Purine/Pyrimidine metabolism and “Terpenoid backbone biosynthesis”. Pentose phosphate pathway and methane metabolism were affected in both Juv_8 and Juv_9. In organismal systems, “PPAR signaling pathway”, “Protein digestion and absorption” and “Cholesterol metabolism” were common to Juv_7 and Juv_9, and “Vitamin digestion and absorption pathway” was shared by Juv_8 and Juv_9. For environmental information processing, Hippo signaling pathway was affected in both Juv_8 and Juv_9. Additionally, specific enriched pathways were found in Juv_7, Juv_8 and Juv_9, respectively.

### 3.5. Differential Gene Expression Analysis in Representative Pathways

**Figure 4:**
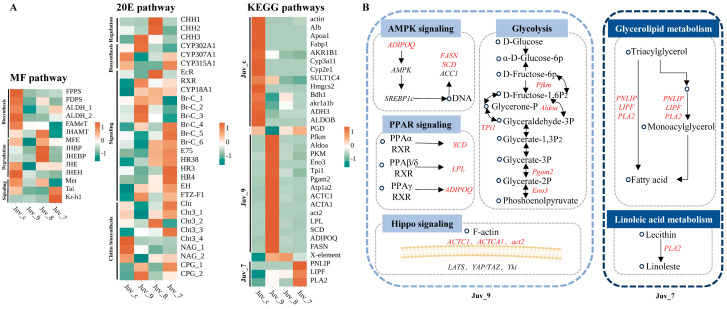
Differential expression analysis of genes in key pathways. (**A**) Heatmap of gene expression in MF, 20E and enriched KEGG pathways. (**B**) Mechanistic diagram of key gene functions. Circles represent substances, italicized text indicates genes, and up-regulated genes are marked in red.

The expression of the MF pathway components was significantly reduced in the experimental group compared to the control group, with the most pronounced effect observed in the Juv_9 group, where all the genes associated with this pathway exhibited down-regulation in expression. Sesquiterpenoid pathway genes (*JHAMT*, *MFE*, *JHBP*, *JHEBP*) were up-regulated in the Juv_8 group. Increased expression of signaling pathway components (*Tai* and *Kr-h1*) in the high-dose group (Juv_7) indicated the response to the dietary MF. The overall response of the 20E pathway was enhanced, including early synthesis gene (*CYP302A1*) and receptor gene (*RXR*) in the Juv_9 group, late synthesis (*CYP307A1*, *CYP315A1*) and signaling (*Br-C*, *E75*, *HR38*, *FTZ-F1*) genes in Juv_7 group, and signaling (*EcR*, *E75*, *HR38*) and crustacean hyperglycemic hormone family (CHH) genes in the Juv_8 group (Figure 4A, Supplementary Tables S4–S6).

Based on the results of the KEGG enrichment analysis, 13 pathways related to growth, metabolism and immunity were identified, with a total of 23 DEGs, which were specifically up-regulated in Juv_c, Juv_9 and Juv_7, respectively (Figure 4A). A total of 13 genes were significantly down-regulated in the experimental group compared to the Juv_c group, mainly related to steroid hormone biosynthesis, glycolysis, etc. Fourteen genes were specifically up-regulated in the Juv_9 group (Figure 4B), involved in AMPK signaling (*ADIPOQ*, *FASN* and *SCD*), PPAR signaling (*SCD*, *LPL* and *ADIPOQ*), Hippo signaling (*ACTC1*, *ACTA1* and *act2*) and Glycolysis pathways (*Pfkm*, *Aldoa*, *Tpi1*, *Pgam2* and *Eno3*). In the Juv_7 group, *PNLIP*, *LIPF* and *PLA2* involved in Glycerolipid metabolism pathway and Linoleic acid metabolism pathway were up-regulated. Furthermore, *PGD* of Pentose phosphate pathway was up- and down-regulated in Juv_7 and Juv_9, respectively, while *X-element* of NF-kappa B signaling pathway was up-regulated in both Juv_9 and Juv_8.

### 3.6. Gene Co-Expression Network

**Figure 5:**
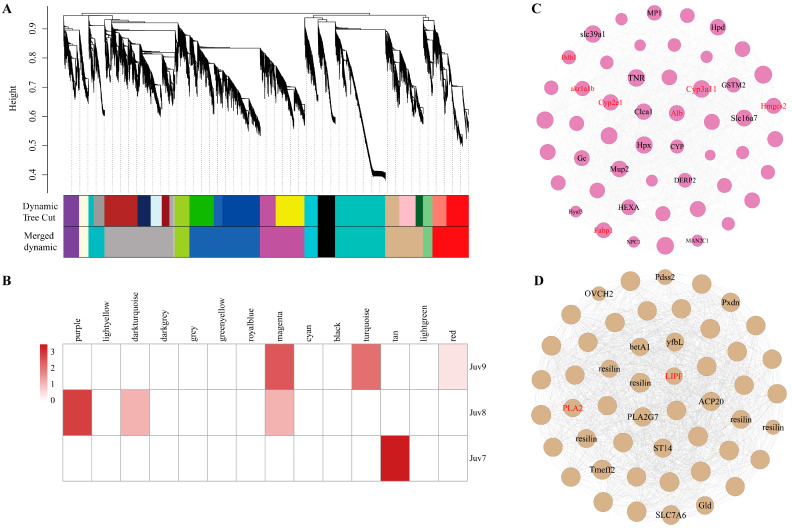
Gene Co-expression Network. (**A**) The WGCNA analysis of *N. denticulata* transcriptome after MF treatment. (**B**) Module–treatment group correlation heatmap. (**C**) Co-expression networks of magenta modules of Juv_9. (**D**) Co-expression networks of tan modules of Juv_7. Annotated transcripts are labeled. Key DEGs in the module are marked in red.

We identified 14 co-expression modules by analyzing the expression correlation of 46,009 genes. Different colors represent different modules (Figure 5A). The largest module was royalblue (including 8023 genes) and the smallest module was grey (including 165 genes) (Figure 5B, Supplementary Table S7). Sample correlation analysis revealed that 5061 genes in the Juv_9 group were significantly enriched to the magenta module, of which 51 genes were DEGs. Combined with the enrichment results of DEGs in key pathways in the previous section, we clarified that *Cyp3a11*, *Cyp2e1*, *Alb*, *Hmgcs2*, *Fabp1*, *akr1a1b* and *Bdh1* were common candidates of both co-expression networks and KEGG pathway enrichment analysis and thus might serve as hub genes for the Juv_9 group (Figure 5C). A total of 4285 genes significantly enriched to the tan module in the Juv_7 group contained 143 DEGs, which were further analyzed by cross-analysis of co-expression networks and key pathways, identifying *PLA2* and *LIPF* as hub genes (Figure 5D). In contrast, the 1787 genes enriched in the purple module of the Juv_8 group contained only eight DEGs, and their potential hub genes need to be further verified in conjunction with subsequent functional experiments.

### 3.7. Expression Patterns of Candidate Genes

**Figure 6:**
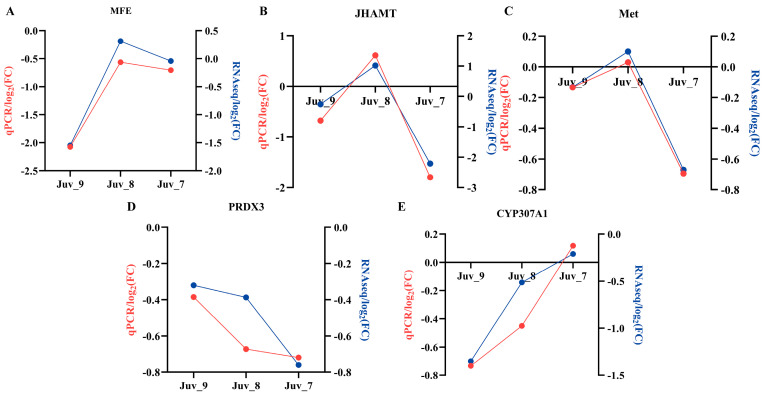
Gene expression validated by RT-qPCR. (**A**) *MFE*; (**B**) *JHAMT*; (**C**) *Met*; (**D**) *PRDX3*; (**E**) *CYP307A1*. Red line represents RT-qPCR results and blue line represents RNA-Seq results.

To validate the accuracy of the RNA-Seq sequencing analysis results, we selected five genes (*MFE*, *JHAMT*, *Met*, *PRDX3* and *CYP307A1*), which are associated with the hormonal pathways for qPCR validation (Figure 6). The gene expression trends revealed by qPCR were consistent with the transcriptome data, thus confirming the accuracy and validity of the transcriptome analysis results. *N. denticulata* treated with different concentrations of MF showed that *MFE*, *JHAMT* and *Met* exhibited a down-regulation of expression in both low- and high-concentrations groups (Juv_9 and Juv_7), while up-regulation was observed in the medium-concentration group (Juv_8). The *PRDX3* showed an enhanced inhibitory effect with increasing MF concentration, whereas the inhibitory effect on the *CYP307A1* weakened as the MF concentration increased, with up-regulation of expression observed in the Juv_7 group.

## 4. Discussion

The growth of crustaceans is influenced by multiple factors, among which endocrine hormones are the main regulating agents [25]. The available literature well represents the direct effect of MF on growth induction in crustaceans, making it a commonly used topical growth promoter [26]. Actually, the influence of MF in regulating growth may vary by species, sex, stage and dosage [7,8,13]. To investigate the underlying mechanisms governing MF-mediated growth regulation, we employed the potential model organism *N. denticulata* to elucidate the dose-dependent regulatory mechanisms of MF during larval development through an integrated analysis of phenotypic traits, physiological parameters and gene expression data.

A study on river prawn larvae not only demonstrated the dose-dependent effect of MF but also revealed its periodic and stage-specific regulatory role in growth [9]. The present study also revealed a dose-dependent effect of MF. Low MF concentrations promoted growth in *N. denticulata*, while high concentrations exhibited inhibitory effects. Notably, the medium-concentration MF exhibited a growth-inhibitory effect initially but switched to growth promotion after 30 days of exposure, further confirming the stage-specific response of organisms to MF. Moreover, studies across multiple species have also demonstrated that the effective physiological range of MF exhibits species and stage-specific characteristics [13].

Digestion is a key part of the growth and metabolic processes in aquatic animals [27]. The activity of digestive enzymes is closely related to digestion and absorption capacity, which determines the nutritional availability for all biological functions [28]. Proteases, amylases and lipases are key digestive enzymes and important physiological indicators related to growth status and stress response [29,30]. In general, animals with higher growth capacity have higher activity of digestive enzymes [31,32]. However, we found that feeding different concentrations of MF resulted in variations in the body length growth rate of *N. denticulata*, the relationship with digestive enzymes does not directly correspond to the growth rate. Additionally, the antioxidant activity of the experimental group was significantly affected, with a remarkable decrease in the activity of SOD and a significant increase in the content of MDA. SOD is an important antioxidant enzyme that catalyzes the conversion of superoxide radicals to hydrogen peroxide, while MDA is a biomarker of oxidative damage caused by lipid peroxidation and its concentration can serve as a biomarker for cellular oxidative status [33,34]. The Juv_8 group exhibited the highest levels of malondialdehyde (MDA), indicating peak lipid peroxidation under this exposure condition. Prolonged stress triggers sustained ROS generation and membrane lipid peroxidation, leading to cellular damage and elevated MDA levels [35]. The shift to growth-promoting effects observed in the Juv_8 group may have contributed to the concurrent accumulation of MDA, suggesting a potential trade-off between growth stimulation and oxidative stress. GSH-Px is another enzymatic antioxidant that scavenges the hydrogen peroxide generated by SOD, thereby contributing to the mitigation of oxidative stress [36]. The increase in GSH-Px activity of Juv_8 and Juv_9 were not statistically significant. However, integrated analysis with other physiological indicators revealed that the organism may initiate compensatory mechanisms. Previous studies have revealed that crustaceans under energy-limited conditions prioritize energy allocation to sustain essential life activities and oxidative stress responses, while compromising energy investment in secondary metabolic pathways [37]. Therefore, our findings suggest that hormonal fluctuations simultaneously induce oxidative stress and growth-related alterations, thereby modulating the organism’s metabolic utilization of nutrients, ultimately leading to a state of relative homeostasis

Recent transcriptomic studies have also shown that MF may regulate the growth and development of crustaceans at the molecular level by controlling the expression of genes related to metabolism, antioxidation and cellular signaling pathways [38]. Our transcriptome data revealed the functional enrichment in each dose group after 40 days feeding period, and dose-dependent GO characteristics were found. Significant enrichment of KEGG pathways involved in muscle development and functions in Juv_9 suggests that low concentrations of MF intake may influence growth and development by regulating genes related to muscle function [39,40]. Enrichment in pathways related to steroidogenesis and body material turnover suggests that the dual-impact on immunity and growth in the medium-concentration group [41,42,43]. In arthropods, distinct classes of chitinases exhibit various biological functions, including the digestion of chitin-rich food, facilitation of molting and participation in immune processes [44,45,46]. Significant enrichment of pathways involved in making the outer skeleton in high MF dose feeding shrimps corroborates these previous studies. Further, specific metabolic and signaling pathways were also affected in each experimental group. This suggests that MF intake could act as a stimulus triggering an immunomodulatory–oxidative stress response and subsequently affecting the digestive processes. On the other hand, each group exhibited distinct pathway enrichments that correlated with their growth and physiological parameter changes. Abundant glucose metabolism in the low dose group enables the organism to potentially maintain a high-energy state, allowing for it to cope with external stress, alleviate metabolic pressure on cardiomyocytes induced by stress, and simultaneously meet the demands of growth promotion [47,48,49,50]. Enrichment of innate immune system-related pathways suggests that middle-dose MF intake shrimps possess a more complex immune stress response mechanism [51,52,53]. Enrichment of pathways related to lipid and some amino acid turnover in the high-dose group probably involved in strategies for regulating lipid metabolism to mitigate excessive immune responses [54,55]. Furthermore, the Juv_9 and Juv_7 groups showed diametrically opposed patterns in growth rate increment compared with controls and Juv_8 exhibited an intermediate phenotype that promotes organismal growth. Therefore, we further conducted pairwise comparative analyses of co-enriched pathways across groups to investigate their regulatory dynamics. Although both Juv7 and Juv_9 showed predominant and significant enrichment in “terpenoid backbone biosynthesis” and “PPAR signaling pathways”, their differential responses to oxidative stress may lead to distinct nutrient metabolism patterns and growth outcomes [56,57,58]. Juv_8 and Juv_9 exhibited enrichment in canonical growth pathways, whereas Juv_8 and Juv_7 showed significant enrichment in hormone-related and immune-associated pathways [59,60,61], suggesting a dose-dependent effect.

To further elucidate the intrinsic relationships among hormones, immune stress, metabolism and growth, we conducted an analysis based on the specific gene expression patterns within relevant pathways. The molting process is crucial for the growth of crustaceans [62]. MF can directly stimulate the Y-organ to secrete ecdysteroids, and the interaction between these two hormonal pathways has been discussed [63]. The MF and ecdysone pathways coordinately participate in the precise regulation of molting. In this study, we observed patterns in the expression changes of genes related to both pathways after MF administration. Compared with the control group, the MF pathway response in the experimental groups showed relative attenuation, with the most pronounced effect observed in the Juv_9 group. In Juv_7, the up-regulated expression of representative signaling pathway genes *Tai* and *Kr-h1* indicates an active MF pathway response. Research indicates that MF exhibits growth-inhibitory effects on crustacean larvae following pathogen challenge, manifested by up-regulation of *JHBP* in both hemolymph and target organs [64]. This enhanced *JHBP* expression accelerates biosynthetic pathways, leading to sustained physiological stress that ultimately results in growth suppression [65]. Conversely, the 20E pathway response was generally enhanced. In the low-dose group, this was mainly manifested by high expression of early synthesis genes and receptor genes, while the high-dose group exhibited elevated expression of late synthesis genes and overall signaling pathway genes. The medium-dose group primarily showed up-regulated expression of signaling pathway genes and *CHHs*. The observed growth retardation may be attributed to molting inhibition, where high dose of exogenous substances potentially induce growth suppression through the overexpression of exoskeleton-related genes [66].

The pathway analysis of DEGs revealed that MF feeding induced changes in nutrient metabolism, similar to a previous report with MF injection [10], primarily reflected in lipid and carbohydrate metabolism. However, the specific response mechanisms differed. AMPK is a crucial kinase regulating energy homeostasis and is closely associated with carbohydrate and lipid metabolism. Previous studies have shown that, in non-nucleic acid-dependent regulation, hormones can participate in “AMPK signaling” by modulating Ca^2+^ to balance carbohydrate and lipid metabolism [67]. The significant up-regulation of genes involved in fatty acid turnover in Juv_9 indicates metabolic reprogramming in the organism. In addition, up-regulation of *SCD*, *LPL* and *ADIPOQ* may be under the control of PPAR receptors (KEGG map03320) [68,69]. PPARα, PPARδ and PPARγ are transcription factors that regulate gene expression upon ligand activation. PPARs also serve as physiological master switches in the heart, controlling cardiac energy metabolism in cardiomyocytes [70,71]. In the “Pentose phosphate pathway”, *pgd* was down-regulated only in Juv_9, suggesting a unique nutrient metabolism pattern in the low-concentration group [72]. Key glycolytic genes were highly expressed in Juv_9, providing the organism with sufficient energy to support growth [47]. This growth-promoting effect was reflected by the up-regulation of act family in the “Hippo pathway”, which may also be closely linked to the “ECM-receptor interaction” [73]. Up-regulation of some genes involved in the “Glycerolipid metabolism pathway” in Juv_7 participates in the direct or indirect conversion of triacylglycerol to fatty acids, indicating enhanced lipid catabolism under high-concentration MF stimulation, presenting a certain pathological state [74]. “Linoleic acid metabolism” is a typical oxidative stress response pathway, and the up-regulation of *PLA2* in Juv_7, promoting the conversion of lecithin to linoleate, suggests intense oxidative stress in the organism [75]. According to WGCNA analysis, hub genes of “Butanoate metabolism”, “Glycolysis”, “Steroid hormone biosynthesis”, “Hippo signaling pathway” and “PPAR pathway” pathways were identified, The “NF-kappa B pathway” regulates the organism’s response to external stimuli, and the elevated expression of *X-element* in medium- and high-dose groups may alleviate oxidative stress [76]. In Juv_7, *LIPF* in “Glycerolipid metabolism” and *PLA2* in “Linoleic acid metabolism” were identified as hub genes, further confirming the pathway-specific regulatory biases in each group and providing a reference for subsequent experimental validation.

## 5. Conclusions

**Figure 7:**
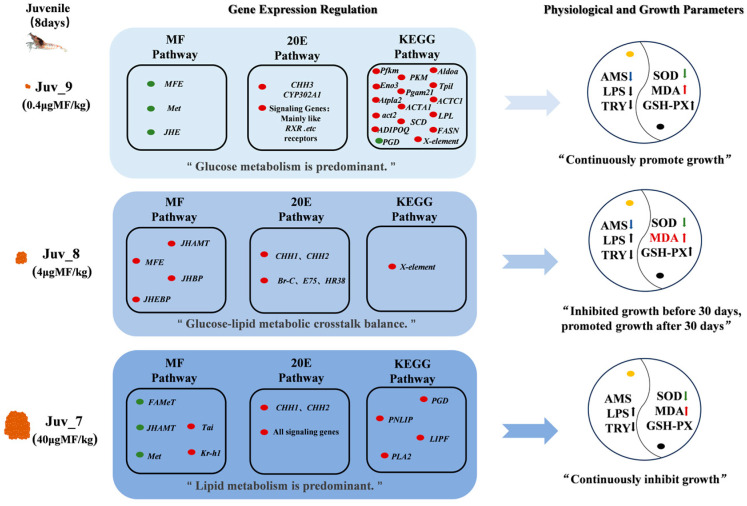
The effects of dietary MF on hormone regulation, oxidative stress, nutrient metabolism, immunity and growth-related processes in juvenile *N. denticulata*. Orange represents MF; red indicates up-regulated genes, while green indicates down-regulated genes; yellow dot denotes digestive enzyme activity, and black dot represents antioxidant indicators; red and green upward arrows indicate a significant up-regulation, whereas blue downward arrows indicate a significant down-regulation; black upward and downward arrows indicate a non-significant up-regulation and down-regulation, respectively.

Our study clarified that MF triggers coordinated changes in hormone regulation, oxidative stress, nutrient metabolism, immunity and growth-related processes in juvenile N. denticulata (Figure 7). The dietary MF directly affects the endocrine hormone response of the shrimp, promoting their growth to a certain extent. Our present study further revealed that a reasonable concentration range, MF promotes the growth of juvenile shrimps through the coordination of endocrine balance and the reprogramming of nutritional metabolism, outlining the intrinsic molecular regulatory mechanisms involved.

## Figures and Tables

**Table 1:** Formulation and proximate composition of experimental feeds (air dry basis, g/kg).

**Ingredients**	**Composition(g/kg)**
Fish meal	400
Soybean meal	270
Wheat flour	220
Fish oil	26
Squid meal	30
Soybean lecithin	10
Yeast extract	24
Vitamin premix	10
Mineral premix	10
Total	1000
**Approximate composition of nutrition level (%)**	
Crude protein	43.14
Crude lipid	8.13
Ash	11.73
Moisture	9.53
**Group**	**MF concentration (μg/kg)**
Juv_9	0.4
Juv_8	4
Juv_7	40

**Table 2:** Primers for quantitative real-time PCR validation.

Gene	Gene Accession Number	Primer Sequence (5′-3′)
EF-1α-F	TRINITY_DN1325_c0_g1	ATTCTCCTGTGCTCGACTGC
EF-1α-R		AGCTCCTTACCAGTACGCCT
MFE-F	TRINITY_DN7155_c0_g1	CTTCCGTCCAGAGCGATTCA
MFE-R		TTCTCCAAGGCACACTCGAC
JHAMT-F	AIY69118.1,TRINITY_DN16742_c0_g1	TTGGATGTGGATCAGGCGAC
JHAMT-R		AGGCTAAGGTGTTGTGCTGA
Met-F	AIY69134.1,TRINITY_DN8620_c0_g1	CCACACACAGGTTGACTTGC
Met-R		GCCTTCTCCATCATGCGACA
PRDX3-F	TRINITY_DN4932_c0_g1	GTGTCCAACTGAGTTAATTGCC
PRDX3-R		GCCTCCTTGCTTCCTTGACA
CYP307A1-F	AIY69127.1,TRINITY_DN47025_c0_g2	TTCGGCTCGTACCTGAACAC
CYP307A1-R		GCATAGCAAAGCAGGCATCC

**Table 3:** Effects of different concentrations of MF on the growth performance of juvenile.

Measurement Indicators	Days	Juv_c	Juv_9	Juv_8	Juv_7
L_0_ (µm)	10	4500 ± 214.7	4388 ± 128.2	4683 ± 186.2	4627 ± 194.4
L_t_ (µm)	20	5038 ± 132.7	4968 ± 101.4	5122 ± 156.5	5057 ± 132.8
	30	6015 ± 217.3	5909 ± 159.7	6089 ± 195.1	6002 ± 167.2
	40	6708 ± 369.8	6671 ± 197.1	6814 ± 304.0	6499 ± 289.4
LGR (%)	20	11.96	13.22	9.37	9.29
	30	19.39	18.94	18.88	18.69
	40	11.52	12.90	11.91	8.28

## Data Availability

The datasets supporting this article are included within the article and its Supplementary Information. Other details will be made available on request.

## Supplementary Materials

The following supporting information can be downloaded at: https://www.mdpi.com/article/10.3390/antiox14060635/s1, Table S1: Summary of Illumina RNA-Seq data; Table S2: GO analysis of DEGs; Table S3: KEGG analysis of DEGs; Table S4: Information of genes in MF pathway; Table S5: Information of genes in 20E pathway; Table S6: Information of genes in KEGG pathways; Table S7: FDR of modules and module size by WGCNA analysis in *Neocaridina denticulata* after MF treatment.

## References

[1] Cheong S.P., Huang J., Bendena W.G., Tobe S.S., Hui J.H. (2015). Evolution of Ecdysis and Metamorphosis in Arthropods: The Rise of Regulation of Juvenile Hormone. Integr. Comp. Biol..

[2] Campli G., Volovych O., Kim K., Veldsman W.P., Drage H.B., Sheizaf I., Waterhouse R.M. (2024). The moulting arthropod: A complete genetic toolkit review. Biol. Rev. Camb. Philos. Soc..

[3] Laufer H., Borst D., Baker F.C., Reuter C.C., Tsai L.W., Schooley D.A., Sinkus M. (1987). Identification of a juvenile hormone-like compound in a crustacean. Science.

[4] Simões L.A.R., Normann R.S., Chung J.S., Vinagre A.S. (2024). A brief and updated introduction to the neuroendocrine system of crustaceans. Mol. Cell Endocrinol..

[5] Nagaraju G.P.C. (2007). Is methyl farnesoate a crustacean hormone?. Aquaculture.

[6] Lovett D.L., Verzi M.P., Clifford P.D., Borst D.W. (2001). Hemolymph levels of methyl farnesoate increase in response to osmotic stress in the green crab, *Carcinus maenas*. Comp. Biochem. Physiol. A Mol. Integr. Physiol..

[7] Homola E., Chang E.S. (1997). Methyl Farnesoate: Crustacean Juvenile Hormone in Search of Functions. Comp. Biochem. Physiol. B Biochem. Mol. Biol..

[8] Wilder M.N., Okada S., Fusetani N., Aida K.J.F.S. (1995). Hemolymph Profiles of Juvenoid Substances in the Giant Freshwater Prawn *Macrobrachium rosenbergii* in Relation to Reproduction and Molting. Fish Sci..

[9] Abdu U., Takac P., Laufer H., Sagi A. (1998). Effect of Methyl Farnesoate on Late Larval Development and Metamorphosis in the Prawn *Macrobrachium rosenbergii* (Decapoda, Palaemonidae): A Juvenoid-like Effect?. Biol. Bull..

[10] Yang Z., Yang X., Du J., Wei C., Liu P., Hu J., Qu Z. (2024). Comparative Transcriptome Analysis of Hepatopancreas Reveals Sexual Dimorphic Response to Methyl Farnesoate Injection in *Litopenaeus vannamei*. Int. J. Mol. Sci..

[11] Møller O., Anger K., Guerao G. (2020). Patterns of Larval Development. Nat. Hist. Crustac..

[12] Lu J., Tao X., Luo J., Zhu T., Jiao L., Jin M., Zhou Q. (2022). Dietary choline promotes growth, antioxidant capacity and immune response by modulating p38MAPK/p53 signaling pathways of juvenile Pacific white shrimp (*Litopenaeus vannamei*). Fish Shellfish Immunol..

[13] Reddy P., Arifullah M. (2021). Dietary methyl farnesoate, a potential growth inducer in male crab *Oziothelphusa senex senex*. IOP Conf. Ser. Earth Environ. Sci..

[14] Mykles D.L., Hui J.H. (2015). *Neocaridina denticulata*: A Decapod Crustacean Model for Functional Genomics. Integr. Comp. Biol..

[15] Kenny N.J., Sin Y.W., Shen X., Zhe Q., Wang W., Chan T.F., Hui J.H. (2014). Genomic sequence and experimental tractability of a new decapod shrimp model, *Neocaridina denticulata*. Mar. Drugs.

[16] Hu F., Wang Y., Hu J., Bao Z., Wang M. (2023). Comparative study of the impact of dietary supplementation with different types of CpG oligodeoxynucleotides (CpG ODNs) on enhancing intestinal microbiota diversity, antioxidant capacity, and immune-related gene expression profiles in Pacific white shrimp (*Litopenaeus vannamei*). Front. Immunol..

[17] Martin M. (2011). CUTADAPT removes adapter sequences from high-throughput sequencing reads. EMBnet J..

[18] Grabherr M.G., Haas B.J., Yassour M., Levin J.Z., Thompson D.A., Amit I., Regev A. (2011). Full-length transcriptome assembly from RNA-Seq data without a reference genome. Nat. Biotechnol..

[19] Manni M., Berkeley M.R., Seppey M., Zdobnov E.M. (2021). BUSCO: Assessing Genomic Data Quality and Beyond. Curr. Protocol..

[20] Buchfink B., Reuter K., Drost H.-G. (2021). Sensitive protein alignments at tree-of-life scale using DIAMOND. Nat. Methods.

[21] Patro R., Duggal G., Love M.I., Irizarry R.A., Kingsford C. (2017). Salmon provides fast and bias-aware quantification of transcript expression. Nat. Methods.

[22] Love M.I., Huber W., Anders S. (2014). Moderated estimation of fold change and dispersion for RNA-seq data with DESeq2. Genome Biol..

[23] Farhadian M., Rafat S.A., Panahi B., Mayack C. (2021). Weighted gene co-expression network analysis identifies modules and functionally enriched pathways in the lactation process. Sci. Rep..

[24] Rao X., Huang X., Zhou Z., Lin X. (2013). An improvement of the 2ˆ (-delta delta CT) method for quantitative real-time polymerase chain reaction data analysis. Biostat. Bioinform. Biomath..

[25] Aiken D.E. (1969). Photoperiod, endocrinology and the crustacean molt cycle. Science.

[26] Hosamani N., Reddy S.B., Reddy R.P. (2017). Crustacean Molting: Regulation and Effects of Environmental Toxicants. J. Mar. Sci. Res. Dev..

[27] He W., Zhou X.Q., Feng L., Jiang J., Liu Y. (2009). Dietary pyridoxine requirement of juvenile Jian carp (*Cyprinus carpio* var. Jian). Aquacult. Nutr..

[28] Gisbert E., Giménez G., Fernández I., Kotzamanis Y., Estévez A. (2009). Development of digestive enzymes in common dentex Dentex dentex during early ontogeny. Aquaculture.

[29] Sudtongkong C., Thongprajukaew K., Hahor W., Saekhow S. (2019). Ontogenetic development of digestive enzymes and elemental composition of sesarmid crab *Episesarma singaporense*. Fish Sci..

[30] Wang W., Ishikawa M., Koshio S., Yokoyama S., Dawood M.A.O., Hossain M.S., Moss A.S. (2019). Effects of dietary astaxanthin and vitamin E and their interactions on the growth performance, pigmentation, digestive enzyme activity of kuruma shrimp (*Marsupenaeus japonicus*). Aquacult. Res..

[31] Lan X., Peng X., Gao Q., Yang G., Yi S., Tang Q. (2024). Comparative analyses on digestive enzyme activities and intestinal structure of Macrobrachium rosenbergii families with different growth performances. Mar. Fish.

[32] Jung H., Lyons R.E., Hurwood D.A., Mather P.B. (2013). Genes and growth performance in crustacean species: A review of relevant genomic studies in crustaceans and other taxa. Rev. Aquacult..

[33] Wei K., Yang J. (2015). Oxidative damage of hepatopancreas induced by pollution depresses humoral immunity response in the freshwater crayfish *Procambarus clarkii*. Fish Shellfish Immunol..

[34] Martindale J.L., Holbrook N.J. (2002). Cellular response to oxidative stress: Signaling for suicide and survival. J. Cell Physiol..

[35] Géret F., Jouan A., Turpin V., Bebianno M.J., Cosson R.P. (2002). Influence of metal exposure on metallothionein synthesis and lipid peroxidation in two bivalve mollusks: The oyster (*Crassostrea gigas*) and the mussel (*Mytilus edulis*). Aquat. Living Resour..

[36] Nordberg J., Arnér E.S.J. (2001). Reactive oxygen species, antioxidants, and the mammalian thioredoxin system. Free. Radic. Biol. Med..

[37] Lin Z., Wu Z., Huang C., Lin H., Zhang M., Chen M., Ruan S. (2023). Cloning and expression characterization of elongation of very long-chain fatty acids protein 6 (elovl6) with dietary fatty acids, ambient salinity and starvation stress in *Scylla paramamosain*. Front. Physiol..

[38] Tamone S.L., Prestwich G.D., Chang E.S. (1997). Identification and Characterization of Methyl Farnesoate Binding Proteins from the Crab, *Cancer magister*. Gen. Comp. Endocr..

[39] Csapo R., Gumpenberger M., Wessner B. (2020). Skeletal Muscle Extracellular Matrix—What Do We Know About Its Composition, Regulation, and Physiological Roles? A Narrative Review. Front. Physiol..

[40] Purslow P.P., Toldrá F. (2023). Chapter 3—The Structure and Growth of Muscle. Lawrie’s Meat Science.

[41] Storbeck K.H., Schiffer L., Baranowski E.S., Chortis V., Prete A., Barnard L., Shackleton C.H.L. (2019). Steroid Metabolome Analysis in Disorders of Adrenal Steroid Biosynthesis and Metabolism. Endocr. Rev..

[42] Hammond G.L. (2016). Plasma steroid-binding proteins: Primary gatekeepers of steroid hormone action. J. Endocrinol..

[43] Glatz J.F. (2015). Lipids and lipid binding proteins: A perfect match. Prostaglandins Leukot. Essent. Fatty Acids.

[44] Salma U., Uddowla M.H., Kim M., Kim J.M., Kim B.K., Baek H.J., Kim H.W. (2012). Five hepatopancreatic and one epidermal chitinases from a pandalid shrimp (*Pandalopsis japonica*): Cloning and effects of eyestalk ablation on gene expression. Comp. Biochem. Physiol. B: Biochem. Mol. Biol..

[45] Zhang S., Jiang S., Xiong Y., Fu H., Sun S., Qiao H., Gong Y. (2014). Six chitinases from oriental river prawn *Macrobrachium nipponense*: cDNA characterization, classification and mRNA expression during post-embryonic development and moulting cycle. Comp. Biochem. Physiol. B: Biochem. Mol. Biol..

[46] Nikapitiya C., Kim W.S., Park K., Kim J., Lee M.O., Kwak I.S. (2015). Chitinase gene responses and tissue sensitivity in an intertidal mud crab (*Macrophthalmus japonicus*) following low or high salinity stress. Cell Stress Chaperones.

[47] Gupta R., Gupta N., Gupta R., Gupta N. (2021). Glycolysis and Gluconeogenesis. Fundamentals of Bacterial Physiology and Metabolism.

[48] Meyer E.E., Clancy C.E., Lewis T.J. (2021). Dynamics of adrenergic signaling in cardiac myocytes and implications for pharmacological treatment. J. Theor. Biol..

[49] Sainio A., Järveläinen H. (2020). Extracellular matrix-cell interactions: Focus on therapeutic applications. Cell Signal..

[50] Mihaylova M.M., Shaw R.J. (2011). The AMPK signalling pathway coordinates cell growth, autophagy and metabolism. Nat. Cell Biol..

[51] Yun S.H., Sim E.H., Goh R.Y., Park J.I., Han J.Y. (2016). Platelet Activation: The Mechanisms and Potential Biomarkers. Biomed. Res. Int..

[52] Ding L.N., Li Y.T., Wu Y.Z., Li T., Geng R., Cao J., Tan X.L. (2022). Plant Disease Resistance-Related Signaling Pathways: Recent Progress and Future Prospects. Int. J. Mol. Sci..

[53] Liu T., Zhang L., Joo D., Sun S.-C. (2017). NF-κB signaling in inflammation. Signal Transduct. Target. Ther..

[54] Liddle R.A., Said H.M. (2018). Chapter 40—Regulation of Pancreatic Secretion. Physiology of the Gastrointestinal Tract.

[55] Omer E., Chiodi C. (2024). Fat digestion and absorption: Normal physiology and pathophysiology of malabsorption, including diagnostic testing. Nutr. Clin. Pract..

[56] Zhou Q.-C., Shi B., Jiao L.-F., Jin M., Sun P., Ding L.-Y., Yuan Y. (2019). Hepatopancreas and ovarian transcriptome response to different dietary soybean lecithin levels in *Portunus trituberculatus*. Comp. Biochem. Physiol. D Genom. Proteom..

[57] Zhou M., Abbas M.N., Kausar S., Jiang C.-X., Dai L.-S. (2017). Transcriptome profiling of red swamp crayfish (*Procambarus clarkii*) hepatopancreas in response to lipopolysaccharide (LPS) infection. Fish Shellfish Immunol..

[58] Zhang Z., Wu Q.-Y., Ge Y., Huang Z.-Y., Hong R., Li A., Yu H.-L. (2023). Hydroxylases involved in terpenoid biosynthesis: A review. Bioresour. Bioprocess..

[59] Ma S., Meng Z., Chen R., Guan K.L. (2019). The Hippo Pathway: Biology and Pathophysiology. Annu. Rev. Biochem..

[60] Schiffer L., Barnard L., Baranowski E.S., Gilligan L.C., Taylor A.E., Arlt W., Storbeck K.H. (2019). Human steroid biosynthesis, metabolism and excretion are differentially reflected by serum and urine steroid metabolomes: A comprehensive review. J. Steroid Biochem. Mol. Biol..

[61] Wang B., Wu L., Chen J., Dong L., Chen C., Wen Z., Wang D.W. (2021). Metabolism pathways of arachidonic acids: Mechanisms and potential therapeutic targets. Signal Transduct. Target. Ther..

[62] de Oliveira Cesar J.R., Zhao B., Malecha S., Ako H., Yang J. (2006). Morphological and biochemical changes in the muscle of the marine shrimp *Litopenaeus vannamei* during the molt cycle. Aquaculture.

[63] Tamone S., Chang E. (1993). Methyl farnesoate stimulates ecdysteroid secretion from crab Y-Organs. Gen. Comp. Endocr..

[64] de Kort C.A.D., Granger N.A. (1996). Regulation of JH titers: The relevance of degradative enzymes and binding proteins. Arch. Insect Biochem. Physiol..

[65] Tsukimura B., Nelson W.K., Linder C.J. (2006). Inhibition of ovarian development by methyl farnesoate in the tadpole shrimp, *Triops longicaudatus*. Comp. Biochem. Physiol. A Mol. Integr. Physiol..

[66] Chang E., Bruce M., Tamone S. (1993). Regulation of Crustacean Molting: A Multi-Hormonal System. Integr. Comp. Biol..

[67] Garcia D., Shaw R.J. (2017). AMPK: Mechanisms of Cellular Energy Sensing and Restoration of Metabolic Balance. Mol. Cell..

[68] Desvergne B., Wahli W. (1999). Peroxisome proliferator-activated receptors: Nuclear control of metabolism. Endocr. Rev..

[69] Yamauchi T., Nio Y., Maki T., Kobayashi M., Takazawa T., Iwabu M., Kadowaki T. (2007). Targeted disruption of AdipoR1 and AdipoR2 causes abrogation of adiponectin binding and metabolic actions. Nat. Med..

[70] Ahmadian M., Suh J.M., Hah N., Liddle C., Atkins A.R., Downes M., Evans R.M. (2013). PPARγ signaling and metabolism: The good, the bad and the future. Nat. Med..

[71] Montaigne D., Butruille L., Staels B. (2021). PPAR control of metabolism and cardiovascular functions. Nat. Rev. Cardiol..

[72] Rippa M., Giovannini P.P., Barrett M.P., Dallocchio F., Hanau S. (1998). 6-Phosphogluconate dehydrogenase: The mechanism of action investigated by a comparison of the enzyme from different species. Biochim. Biophys. Acta Protein Struct. Mol. Enzymol..

[73] Misra J.R., Irvine K.D. (2018). The Hippo Signaling Network and Its Biological Functions. Annu. Rev. Genet..

[74] Prentki M., Madiraju S.R. (2008). Glycerolipid metabolism and signaling in health and disease. Endocr. Rev..

[75] Mercola J. (2025). Linoleic acid, mitochondria, gut microbiome, and metabolic health: A mechanistic review. Adv. Redox Res..

[76] Guo W., Wang D., Lisch D. (2021). RNA-directed DNA methylation prevents rapid and heritable reversal of transposon silencing under heat stress in Zea mays. PLoS Genet..

